# Impact of two mycotoxins deoxynivalenol and fumonisin on pig intestinal health

**DOI:** 10.1186/s40813-016-0041-2

**Published:** 2016-09-14

**Authors:** Alix Pierron, Imourana Alassane-Kpembi, Isabelle P. Oswald

**Affiliations:** 1 ToxAlim Research Centre in Food Toxicology, INRA, UMR 1331, ENVT, INP Purpan, 180 chemin de Tournefeuille, BP93173, 31027 Toulouse, Cedex 03 France; 2 BIOMIN Research Center, Technopark 1, 3430 Tulln, Austria

**Keywords:** Pig, Fumonisin B_1_, Deoxynivalenol, Feed contamination, Intestine, Barrier function, Immune response

## Abstract

Mycotoxins are secondary metabolites of fungi that grow on a variety of substrates. Due to their high consumption of cereals and their sensitivity, pigs are highly impacted by the presence of mycotoxins. At the European level, regulations and recommendations exist for several mycotoxins in pig feed. Among these toxins, fumonisin B_1_ (FB_1_), and deoxynivalenol (DON) have a great impact on the intestine and the immune system. Indeed, the intestine is the first barrier to food contaminants and can be exposed to high concentrations of mycotoxins upon ingestion of contaminated feed. FB_1_ and DON alter the intestinal barrier, impair the immune response, reduce feed intake and weight gain. Their presence in feed increases the translocation of bacteria; mycotoxins can also impair the immune response and enhance the susceptibility to infectious diseases. In conclusion, because of their effect on the intestine, FB_1_ and DON are a major threat to pig health, welfare and performance.

## Background

Food safety is a major issue throughout the world. In this respect, much attention needs to be paid to the possible contamination of food and feed by fungi and the risk of mycotoxin production. Mycotoxins are secondary metabolites produced by filamentous fungi, mainly by species from the genus *Aspergillus*, *Fusarium* and *Penicillium*. They are produced on a wide variety of substrates before, during and after harvest. Mycotoxins are very resistant to technological treatments and difficult to eliminate; therefore they can be present in human food and animal feed [1]. The ingestion of mycotoxin-contaminated feed can induce acute diseases, and the ingestion of low doses of fungal toxins also causes damage in case of repeated exposure [2, 3].

**Fig. 1:**
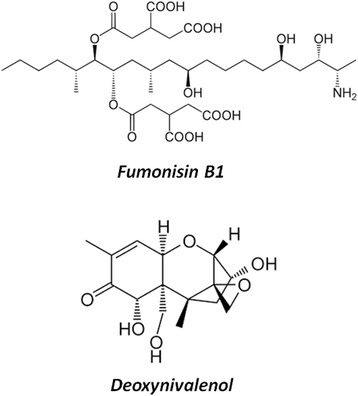
Chemical structure of Fumonisin B_1_ and Deeoxynivalenol. These two mycotoxins belong to different families, with many different chemical structures and so various effects induced

Monogastric livestock, pig and poultry, are particularly vulnerable to mycotoxins because of the high percentage of cereals in their diet and because they lack a rumen with a microbiota able to degrade mycotoxins before their intestinal absorption. From an intestinal pig health perspective, the most notorious mycotoxins (Fig. 1) are fumonisins, especially fumonisin FB_1_ (FB_1_) and trichothecenes, especially deoxynivalenol (DON) [4]. In the European Union, some recommendations exist for both toxins in pig feed (Table 1).

**Table 1:** Recommendations for DON and FB_1_ in pigs feed and feedstuffs. Depending of the mycotoxin and the type of feed intended to pigs, different directive and recommendation exist about the concentration authorized. (EC Recommendations 2006/576/EC and 2013/165/EU)

Mycotoxins	Pig feeds	Max. content mg/Kg (ppm)
DON	Cereals (without maize by-products)	8 (12)
	Complete and complementary feeding stuffs for pigs	0.9
FB_1_ + FB_2_	Cereals	60
	Complete and complementary feeding stuffs for pigs, horse and rabbit	5

This review will summarize the effect of FB_1_ and DON on the intestine and analyze the consequences in terms of pig health.

### Toxicity of DON and FB1

#### Toxicity of DON

DON is a 12,13-epoxy-3α,7α,15-trihydroxytrichothec-9-en-8-on (Fig. 1). Numerous studies bring information on the toxic effects of DON in mamals, especially rodents [5–7]. At the molecular level, DON targets the ribosome. It binds to the A-site of the peptidyl transferase center (PTC) of this organelle [8]. This binding is linked to the epoxy- and C3- group of the DON molecule [9]. Interaction with the ribosome leads to an inhibition of the elongation of chain elongation step of protein synthesis leading to an inhibition of RNA, DNA and protein synthesis [6]. This ribosome binding activates several ribosome-associated mitogen activated protein kinases (MAPKs), including p38, c-Jun N-terminal Kinase (JNK), and extracellular signal-regulated kinase 1 and 2 (ERK1/2), an effect called “ribotoxic stress” response [10].

A high concentration DON causes effects and symptoms similar to those observed during an exposure to ionizing radiation, such as abdominal distress, salivation, discomfort, diarrhea, vomiting, leukocytosis and gastrointestinal bleeding. This mycotoxin also has high emetic and anorexic effects resulting in growth suppression [11, 12]. The colloquial name of DON is “vomitoxin” due to its strong emetic effects observed in pigs [13]. The underlying mechanisms for anorexia are not yet fully understood. Two major mediators of DON-induced anorexia, *i.e*. pro-inflammatory cytokines and satiety hormones, have emerged from studies carried out mainly in mice [10, 14]. It is worth to point out that, contrary to humans or pigs, emesis cannot occur in rodents, but the abnormal food intake behaviour observed in mice (or other rodents) is considered indicative of nausea-induced anorexia [6].

The immune system is sensitive to DON and can be either stimulated or suppressed depending on dose, exposure frequency, timing and the functional immune assay being employed [10]. Leukocytes, most notably mononuclear phagocytes, play a likely central role in the acute and chronic toxicity evoked by DON. Low concentrations of DON induce expression of early response and pro-inflammatory genes at the mRNA and protein levels, while high concentrations promote rapid onset of leukocyte apoptosis. This immune dysregualtion is a consequence of the ribotoxic stress. Indeed, activation of p38 and ERK1/2 triggers two competing signaling pathways, one down-stream of p38 favoring apoptosis and one downstream of ERK1/2 favoring survival and cytokine expression [6]. DON also impairs humoral and cell-mediated responses, alters serum IgA levels, IgA-associated nephropathy [15].

Others studies show, that DON can also have reproductive and teratological effects, with increase of skeletal abnormalities, neural arch defects or fusion, and genotoxic effects with the induction of oxydative stress mediated DNA damage on cells [16]. By contrast, there is inadequate evidence in experimental animals for the carcinogenicity of DON and the International Agency for Research on Cancer (IARC), placed DON in Group 3, “not classifiable as to its carcinogenicity to humans”.

#### Toxicity of FB1

Fumonisin B_1_ (FB_1_) is the diester of propane-1,2,3-tricarboxylic acid and 2-amino-12,16-dimethyl- 3,5,10,14,15-pentahydroxyeicosane (Fig. 1). Its toxicity have been broadly reviewed [17, 18]. The primary amine function and the tricarballylic acid side chains appears necessary for the biological activity of FB_1_, as N-substituted fumonisin and hydrolized fumonisin fail to elicit effects both *in vitro* and *in vivo* [19, 20]. FB_1_ has an unsubstituted primary amino group at C2 and competitively inhibits ceramide synthase, which results in disruption of the *de novo* biosynthesis of ceramide and alteration of the sphingolipid metabolism. An immediate consequence of the ceramide synthase inhibition is accumulation of the enzyme’s substrates sphinganine (Sa) and, to a lesser degree, sphingosine (So) in tissues, serum, and urine. In facts, increase in the Sa:So ratio in tissues and bio-fluids are explored as biomarker to fumonisin exposure in several species though these modifications of sphingoid base profiles are transient [21, 22].

A correlation between the fumonisin-induced Sa accumulation and the onset of apoptosis and mitosis has been shown in the liver and kidney of several species including pig [23, 24]. Moreover, the depletion of specific sphingolipids associated to the membrane lipid rafts involved in folate transport was suggested as the mechanism by which FB_1_ disrupts the 5-methyltetrahydrofolate uptake in cells [25]. The primary consequence of the disrupted folate uptake may be the teratogenic effect reported with FB_1_ given intraperitoneally to pregnant dams leading to neural tube defects in embryo [26]. Folate deficiency as a risk factor for neural tube defects is well established [27]. Besides the neural tube defects in newborns, the symptoms induced by FBs are unusually broad and include, brain lesions in horses, lung edema in swine as well as cancer in experimental animals. The International Agency for Research on Cancer (IARC) classified FB_1_ in Group 2B as ‘possibly carcinogenic to humans’.

Especially in pigs, fumonisins are poorly absorbed from the gastrointestinal tract. The calculated bioavailability for FB_1_ was approximately 0.041 of the dose [28]. The absorbed fraction remains in the tissues (preferentially in liver and kidneys) for an extended period of time, and enterohepatic recirculation contributes to the long biological half-life of the mycotoxin [28, 29].

The fumonisin toxicosis in pig is well documented. Historically, outbreaks of a fatal disease in pigs fed *Fusarium verticillioides*-contaminated maize crop in mid-western and south-eastern USA in 1989 led to the identification of FB_1_ as the causative agent of porcine pulmonary edema (PPE) [30]. Within 4–7 days of initial feeding of highly contaminated feed, pigs show respiratory distress and cyanosis that is rapidly followed by death due to acute pulmonary edema and hydrothorax [31]. Non-lethal pulmonary edema has also been reported following longer term, lower dose exposures [32]. The fumonisin-induced pulmonary edema appears to result from acute left-sided heart failure, as FB_1_ has been shown to decrease cardiac contractility, mean systemic arterial pressure, heart rate and cardiac output, and increases mean pulmonary artery pressure and pulmonary artery wedge pressure [33, 34]. This cardiotoxicity was also documented in horse following intraveinous administration of purified FB_1_ [35].

Additional findings reported in pig from chronic exposure studies include right ventricular hypertrophy due to pulmonary hypertension, hepatic injury characterized by icterus with severe hepatic fibrosis and nodular hyperplasia and effects on both specific and non-specific immunity [36, 37]. FB_1_ decreased phagocytosis and inhibited sphingolipid biosynthesis in pig pulmonary macrophages, and decreased clearance of particles and bacteria from the pulmonary circulation [38, 39].

Regarding the immunity, dietary exposure to FB_1_, even at low doses is associated to sex-specific decrease of antibody titers following vaccination and increased swine susceptibility to opportunistic pathogens [40, 41]. Of note, gender-dependent immunosuppression following subacute exposure to FB_1_ has also been described in mice, and the authors hypothetized that the selective alterations in lymphocyte functions and dramatic reduction in specific thymocytes in females may be related to FB_1_-induced alterations in estrogen metabolism and signaling [42].

### Effects of DON and FB1 on the pig intestine

The toxicity of DON and FB_1_ varies according to several parameters such as the dose, the duration of exposure, the age and the sex of the animal, as well as nutritional factors [43–45]. Their effects on performance are greater in males and young pigs [41, 45].

The intestinal tract is the first target for mycotoxins following ingestion of contaminated feed. The intestinal epithelium is a single layer of cells lining the gut lumen that acts as a selective filter, allowing the absorption of dietary nutrients, essential electrolytes, and water from the intestinal lumen into the blood circulation [46]. It also constitutes the largest and most important barrier to prevent the passage of harmful intraluminal substances from the external environment into the organism, including foreign antigens, microorganisms, and their toxins [47, 48]. Following the ingestion of mycotoxin-contaminated feed, intestinal epithelial cells may be exposed to high concentrations of toxins, potentially affecting intestinal functions [49–51].

#### Effect on Feed intake

DON and to a letter extend FB_1_ have an effect on feed intake and subsequent animal growth.

The colloquial name of DON, vomitoxin, refers to its emetic effect observed both in field reports and in experimental intoxications where high doses of the toxin were given orally or intravenously to pigs. Complete feed refusal was observed at levels of 12 and vomiting at 20 mg DON/kg feed. Pig feeding trials with naturally or artificially contaminated diets have shown decreased feed consumption and weight gain at doses from 0.6 to 3 mg DON/kg feed [52]. A meta-analysis showed that deoxynivalenol reduced feed intake and weight gain by 26 %; the same analysis also demonstrated a 16 % reduction of feed intake in response to aflatoxin B_1_ (AFB_1_) [45].

Consumption of pure FB_1_ or FB_1_-contaminated feed also induces a slight reduction of feed intake and body weight in piglets. Although FB_1_ is poorly absorbed and metabolized in the intestine, it induces intestinal disturbances (abdominal pain or diarrhea) and cause extra-intestinal organ pathologies [53].

#### Effect on intestinal digestion and nutrient absorption

At the molecular level DON and FB_1_ have been shown to alter the absorptive functionality of the intestine.

The sodium-glucose dependent transporter (SGLT-1) activity is particularly sensitive to DON. SGLT-1 is the main apical transporter for active glucose uptake in the small intestine [54]. Inhibition of SGLT-1 by DON has nutritional consequences and could explain diarrhea associated with DON ingestion, since this transporter is responsible for daily absorption of water in the gut [5]. DON not only impairs the intestinal absorption of sugars (glucose and fructose), but also alters the uptake of palmitate and monocarboxilates in the jejunum [55].

In contrast to DON, sodium-dependent glucose absorption is up-regulated in pig after acute or long term exposure to FB_1_ [56, 57]. Pigs consuming corn culture extracts containing FB also showed a markedly lowered activity of aminopeptidase N [56]. Likewise, exposure to 1.5 mg/kg b.w. FB_1_ has been shown to induce sphingolipid depletion in pig intestinal epithelium, which can result in a deficiency of folate uptake [50, 58].

#### Effect on intestinal histomorphology

Consumption of mycotoxin-contaminated feed induces histological damage on intestinal tissue. Epithelial lesions (multifocal atrophy, villi fusion, apical necrosis of villi, vacuolation of enterocytes and edema of lamina propria) in the intestine of pigs fed with a diet naturally contaminated with DON have been observed [52, 59]. No effect was observed on crypt depth. Jejunal lesions, including shortened and coalesced villi, lysis of enterocytes, and edema, were also observed in an *ex-vivo* model of intestinal tissues after exposure to DON [60–62]. Exposure to FB also induces changes in intestinal villi morphology such as reduced villi height and villi fusion and atrophy [52]. As described in poultry, the morphological changes may lead to a decrease of nutrients absorption by enterocytes, a reduced energy and nutrient uptake and impaired growth [63].

#### Effect on barrier function

Both DON and FB_1_ alter intestinal barrier functions. Several studies have investigated the effect of DON on the transepithelial electrical resistance (TEER), a good indicator of the integrity of the barrier function. DON decreases TEER in pig intestinal epithelial cells in a time and dose dependant manner [9, 51, 60, 64]. In piglets jejunal explants the paracellular passage, assessed in Ussing chambers, was significantly increased in presence of 20 to 50 μM of DON [65]. Similarly to DON, FB_1_ impaired the integrity of porcine intestinal epithelial cell line derived from the jejunum (IPEC-J2) monolayer via altered viability and reduced TEER [66]. It has also been observed that a prolonged exposure to FB_1_ prevents the establishment of the TEER and alters the resistance of an already established monolayer of porcine intestinal epithelial cells [67].

At the molecular level, these toxins affect the intestinal epithelium permeability through modulation of the tight junction complexes [50, 51]. A defective expression of occludin and E-cadherin has been observed in the ileum of piglets fed low doses of FB_1_ [61]. The FB-induced alteration of the sphingolipid biosynthesis pathway and the associated lipid rafts could also contribute to impairing the establishment and maintenance of tight junctions [53]. Likewise, the activation of MAPKs by DON affects the expression and cellular localization of proteins forming or being associated with tight junctions such as claudins and ZO-1, which results in increased intestinal paracellular permeability [60].

The loss of tight junction integrity and resulting increased paracellular permeability may lead to increased bacterial translocation across the intestine and increased susceptibility to enteric infections. Such an increase in bacterial passage through intestinal epithelial cells has major implications for pig health in terms of sepsis, inflammation and enteric infection.

Differentiated IPEC-J2 cells treated 24 h with 0.1-10 μM DON in a co-exposure with *Salmonella* Typhimurium bacteria show a significant increase of the translocation of the bacteria across intestinal epithelial cells [68]. On differentiated IPEC-1 cells treated 48 h with DON an increase translocation of *Escherichia coli* was observed in 17, 50 and 63 % with 5, 10 and 20 μM DON respectively [65]. So, DON is able to increase the passage of macromolecule and bacteria in intestinal epithelial cells.

Two separate studies analyzed the effect of low to moderate doses of FB_1_ on intestinal colonization and mucosal response to pathogenic strains of *E. coli* [69, 70]. They both demonstrated a higher susceptibility of intestinal *E. coli* infection of piglets exposed to the toxin. Translocation of bacteria to the mesenteric lymph nodes and dissemination to the lungs, and to a lesser extent to liver and spleen, were observed in FB_1_-treated pigs in comparison to untreated animals [70].

#### Modulation of intestinal immune response

DON and FB_1_ impact the systemic and/or the local immune response (review [5, 10, 53]). As far as pig is concerned, several studies have investigated the effect of theses mycotoxins on the intestinal immune system.

The effect of ingestion of FB_1_ was measured on the intestinal production of 5 inflammatory cytokines (IL-1β, IL-6, IL-12, TNF-β and IL-8). Both *in vitro* and *in vivo* data indicate that FB_1_ specifically decreases expression of IL-8 mRNA [71]. IL-8 being involved in the recruitment of inflammatory cells in the intestine during infection [72–74], this specific decrease of intestinal IL-8 may contribute to the observed increased susceptibility of FB_1_-treated piglets to *E. coli* infection [70]. The increased susceptibility to intestinal infection is also correlated with a reduced intestinal expression of IL-12p40, an impaired function of intestinal antigen presenting cells (APC), a decreased upregulation of Major Histocompatibility Complex Class II molecule (MHC-II) and reduced T cell stimulatory capacity [69].

DON modulates intestinal immunity both directly (through activation of signalling pathways) and indirectly (through crossing of luminal bacterial antigens, which was observed together with bacterial translocation following mucus layer alteration and tight junction opening) [75]. In a pig jejunal explant model, DON has been shown to trigger the innate as well as adaptative immunity [76]. Intestinal exposure to DON induced a pro-inflammatory response with a significant increase of expression of TNF-α, IL-1α, IL-1β, and IL-8. Moreover, DON up-regulated the expression of genes involved in the differentiation of Th17 cells (STAT3, IL–17A, IL-6, IL-1b) at the expenses of the pathway of regulatory T cells (FoxP3, RALDH1). DON also induced genes related to the pathogenic Th17 cells subset such as IL–23A, IL-22 and IL-21 and not genes related to the regulatory Th17 cells such as TGF-b and IL-10 [76]. Likewise, DON potentiated the up-regulation of IL-1β, IL-8, MCP1 and IL-6 induced by *S.* Typhimurium in pig intestinal loops [68].

#### Intestinal microbiota

As other fungi secondary metabolites especially antibiotics, several mycotoxins have demonstrated antimicrobial properties [77, 78]. As a consequence, mycotoxins may modify the intestinal microflora. Surprisingly, this impact of mycotoxins has been poorly investigated. Two studies have investigated the impact of DON and FB_1_ on the intestinal microflora [79, 80].

The first study investigated the impact of DON on the intestinal microflora by Capillary Electrophoresis Single-Stranded Conformation Polymorphism (CE-SSCP). Consumption of feed naturally contaminated with DON (2.8 mg/kg feed) for four weeks had a moderate effect on total faecal Aerobic Mesophilic Bacteria and Anaerobic Sulfite-Reducing. By constrast, DON changed the faecal microflora balance; it did not impact the diversity index but modulate the richness index [79].

In the second study, pigs received feed contaminated with 12 mg FB/kg feed for 63 days. This diet transiently affected the balance of the digestive microbiota during the first four weeks of exposure as measured by SSCP feacal microbiota profiles; a co-infection with *S.* typhimurium amplified this phenomenon and change the microbiota profile. As already observed with DON, aerobic mesophylic bacteria count was not change by FB_1_ treatment [80].

## Conclusion

**Fig. 2:**
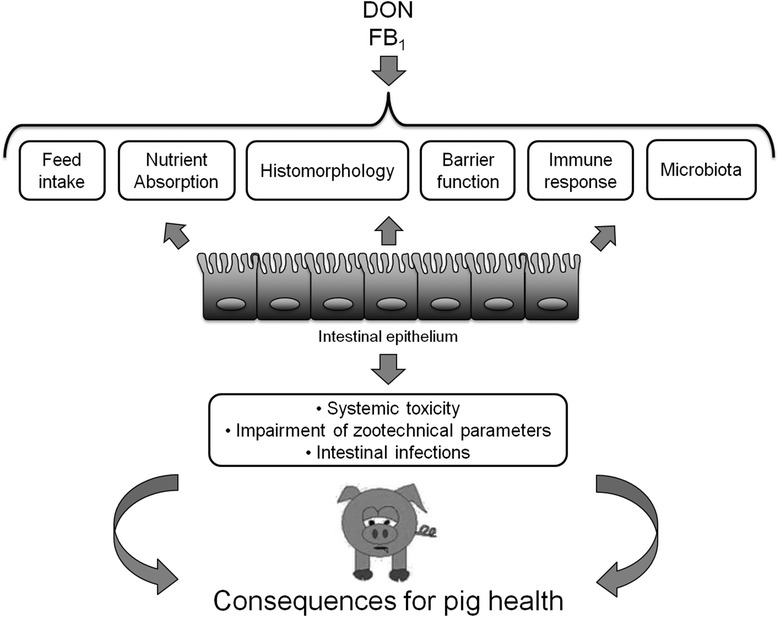
Summary of the intestinal toxicity of the main mycotoxins present in feed pig. DON and FB_1_ can induce several effects on the intestine, with at the end a global impact on the pig intestinal health

Regulations and recommendations exist for six mycotoxins (AF, FB, Ochratoxin A (OTA), zearalenone (ZEN), T2/HT2 toxins (T2/HT2) and DON) present in pig feed. Among them, DON and FB have been studied for their toxicity in the intestine of pig. The intestine is a target for mycotoxins and as illustrated in this paper, the fact that the intestine is a target for DON and FB_1_ have some consequences in terms of pig health (Fig. 2). Theses mycooxins are not only locally toxic for the intestine, but also dysregulate many intestinal functions and impair the local immune response. This results in systemic toxicity leading to many symptoms, alteration of zootechnical parameters. Feed contamination with mycotoxins also increases impair the barrier function of the intestine, leading to translocation of bacteria across the intestine and thus intestinal and systemic infections.

Global surveys indicate that animals are generally exposed to more than one mycotoxin [81]. Indeed fungi are able to produce several mycotoxins simultaneously; and it is common practice to use multiple grains in animal diets. Unfortunately, the toxicity of mycotoxin mixtures cannot be predicted based on their individual toxicities. Interactions between concomitantly occurring mycotoxins can be antagonistic, additive, or synergistic [82]. The data on combined toxicity of mycotoxins are limited and therefore, the health risk from exposure to a combination of mycotoxins is incompletely understood [83, 84] and deserves further investigation.

## Abbreviations

AFB_1_, Aflatoxin B_1_; AFB_2_, Aflatoxin B_2_; CE-SSCP, Capillary Electrophoresis Single-Stranded Conformation Polymorphism; DNA, Deoxyribonucleic acid; DON, Deoxynivalenol; ERK 1/2, Extracellular Signal Regulated Kinase 1 and 2; FB_1_, Fumonisin B_1_; FoxP3, Forkhead box P3; HT2, HT2 toxin; IARC, International Agency for Research on Cancer; Ig, Imunoglobulin; IL, Interleukin; IPEC-J2, Porcine Intestinal Epithelial Cell line derived from the jejunum; JNK, C-Jun N-terminal Kinase; MAPKs, Mitogen Activated Protein Kinases; MCP1, Monocyte chemoattractant protein-1; MHC-II, Major Histocompatibility Complex - Class II; OTA, Ochratoxin A; OVA, Ovalbumin; PPE, porcine pulmonary edema ; PTC, Peptidyl Transferase Center; RALDH1, Retinaldehyde dehydrogenase 1; RNA, Ribonucleic acid; Sa, Sphinganine; SGLT-1, Sodium-glucose dependent transporter; So, Sphingosine; STAT3, Signal transducer and activator of transcription 3; T2, T2 toxin; TEER, Trans Epithelial Electrical Resistance; TGF, Transforming Growth Factor; TNF, Tumor Necrosis Factor; ZEN, Zearalenone

## References

[1] Hazel CM, Patel S (2004). Influence of processing on trichothecene levels. Toxicol Lett.

[2] Bryden WL (2012). Mycotoxin contamination of the feed supply chain : Implications for animal productivity and feed security. Anim Feed Sci Technol.

[3] Maresca M, Fantini J (2010). Some food-associated mycotoxins as potential risk factors in humans predisposed to chronic intestinal inflammatory diseases. Toxicon.

[4] CAST (2003). Potential economic costs of mycotoxins in United States. Task Force Report 138. Mycotoxins: Risks in plant, animal and human systems.

[5] Maresca M (2013). From the gut to the brain: journey and pathophysiological effects of the food-associated mycotoxin Deoxynivalenol. Toxins.

[6] Pestka JJ (2010). Deoxynivalenol: mechanisms of action, human exposure, and toxicological relevance. Arch Toxicol.

[7] Wang Z, Wu Q, Kuca K, Dohnal V, Tian Z (2014). Deoxynivalenol: signaling pathways and human exposure risk assessment--an update. Arch Toxicol.

[8] Garreau de Loubresse N, Prokhorova I, Holtkamp W, Rodnina MV, Yusupova G, Yusupov M (2014). Structural basis for the inhibition of the eukaryotic ribosome. Nature.

[9] Pierron A, Mimoun S, Murate LS, Loiseau N, Lippi Y, Bracarense A-PFL (2016). Microbial biotransformation of DON: molecular basis for reduced toxicity. Sci Rep.

[10] Pestka JJ (2010). Deoxynivalenol-induced proinflammatory gene expression: mechanisms and pathological sequelae. Toxins (Basel).

[11] Pestka JJ, Smolinski AT (2005). Deoxynivalenol: toxicology and potential effects on humans. J Toxicol Environ Health B Crit Rev.

[12] Haschek WM, Voss KA, Beasley V, Haschek WM, Rousseaux CG, Wallig MA (2002). Selected mycotoxins affecting animal and human health. Handbook of Toxicological Pathology.

[13] Vesonder RF, Ciegler A, Jensen AH (1973). Isolation of the emetic principle from Fusarium-infected corn. Appl Microbiol.

[14] Lebrun B, Tardivel C, Felix B, Abysique A, Troadec JD, Gaige S (2015). Dysregulation of energy balance by trichothecene mycotoxins: Mechanisms and prospects. Neurotoxicology.

[15] Sobrova P, Adam V, Vasatkova A, Beklova M, Zeman L, Kizek R (2010). Deoxynivalenol and its toxicity. Interdiscip Toxicol.

[16] Sun XM, Zhang XH, Wang HY, Cao WJ, Yan X, Zuo LF (2002). Effects of sterigmatocystin, deoxynivalenol and aflatoxin G1 on apoptosis of human peripheral blood lymphocytes in vitro. Biomed Environ Sci.

[17] Escriva L, Font G, Manyes L (2015). In vivo toxicity studies of fusarium mycotoxins in the last decade: a review. Food Chem Toxicol.

[18] Voss KA, Smith GW, Haschek WM (2007). Fumonisins: Toxicokinetics, mechanism of action and toxicity. Anim Feed Sci Tech.

[19] Howard PC, Couch LH, Patton RE, Eppley RM, Doerge DR, Churchwell MI (2002). Comparison of the toxicity of several fumonisin derivatives in a 28-day feeding study with female B6C3F(1) mice. Toxicol Appl Pharm.

[20] Grenier B, Bracarense AP, Schwartz HE, Trumel C, Cossalter AM, Schatzmayr G (2012). The low intestinal and hepatic toxicity of hydrolyzed fumonisin B_1_ correlates with its inability to alter the metabolism of sphingolipids. Biochem Pharmacol.

[21] Enongene EN, Sharma RP, Bhandari N, Miller JD, Meredith FI, Voss KA (2002). Persistence and reversibility of the elevation in free sphingoid bases induced by fumonisin inhibition of ceramide synthase. Toxicol Sci.

[22] Voss KA, Plattner RD, Riley RT, Meredith FI, Norred WP (1998). In vivo effects of fumonisin B(1)-producing and fumonisin B(1)-nonproducing Fusarium moniliforme isolates are similar: Fumonisins B(2) and B(3) cause hepato- and nephrotoxicity in rats. Mycopathologia.

[23] Gumprecht LA, Beasley VR, Weigel RM, Parker HM, Tumbleson ME, Bacon CW (1998). Development of fumonisin-induced hepatotoxicity and pulmonary edema in orally dosed swine: morphological and biochemical alterations. Toxicol Pathol.

[24] Gumprecht LA, Marcucci A, Weigel RM, Vesonder RF, Riley RT, Showker JL (1995). Effects of intravenous fumonisin B1 in rabbits: nephrotoxicity and sphingolipid alterations. Nat Toxins.

[25] Stevens VL, Tang J (1997). Fumonisin B1-induced sphingolipid depletion inhibits vitamin uptake via the glycosylphosphatidylinositol-anchored folate receptor. J Biol Chem.

[26] Gelineau-van Waes J, Starr L, Maddox J, Aleman F, Voss KA, Wilberding J (2005). Maternal fumonisin exposure and risk for neural tube defects: mechanisms in an in vivo mouse model. Birth Defects Res A Clin Mol Teratol.

[27] Pitkin RM (2007). Folate and neural tube defects. Am J Clin Nutr.

[28] Prelusky DB, Trenholm HL, Rotter BA, Miller JD, Savard ME, Yeung JM (1996). Biological fate of fumonisin B1 in food-producing animals. Adv Exp Med Biol.

[29] Prelusky DB, Trenholm HL, Savard ME (1994). Pharmacokinetic fate of 14C-labelled fumonisin B1 in swine. Nat Toxins.

[30] Osweiler GD, Ross PF, Wilson TM, Nelson PE, Witte ST, Carson TL (1992). Characterization of an Epizootic of Pulmonary-Edema in Swine Associated with Fumonisin in Corn Screenings. J Vet Diagn Invest.

[31] Haschek WM, Motelin G, Ness DK, Harlin KS, Hall WF, Vesonder RF (1992). Characterization of Fumonisin Toxicity in Orally and Intravenously Dosed Swine. Mycopathologia.

[32] Zomborszky-Kovacs M, Vetesi FF, Kovacs F, Bata A, Toth A, Tornyos G (2000). Preliminary communication: Examination of the harmful effect to fetuses of fumonisin B-1 in pregnant sows. Teratogen Carcin Mut.

[33] Constable PD, Smith GW, Rottinghaus GE, Tumbleson ME, Haschek WM (2003). Fumonisin-induced blockade of ceramide synthase in sphingolipid biosynthetic pathway alters aortic input impedance spectrum of pigs. Am J Physiol Heart Circ Physiol.

[34] Smith GW, Constable PD, Tumbleson ME, Rottinghaus GE, Haschek WM (1999). Sequence of cardiovascular changes leading to pulmonary edema in swine fed culture material containing fumonisin. Am J Vet Res.

[35] Smith GW, Constable PD, Foreman JH, Eppley RM, Waggoner AL, Tumbleson ME (2002). Cardiovascular changes associated with intravenous administration of fumonisin B-1 in horses. Am J Vet Res.

[36] Casteel SW, Turk JR, Cowart RP, Rottinghaus GE (1993). Chronic Toxicity of Fumonisin in Weanling Pigs. J Vet Diagn Invest.

[37] Harvey RB, Edrington TS, Kubena LF, Elissalde MH, Rottinghaus GE (1995). Influence of Aflatoxin and Fumonisin B-1-Containing Culture Material on Growing Barrows. Am J Vet Res.

[38] Haschek WM, Gumprecht LA, Smith G, Tumbleson ME, Constable PD (2001). Fumonisin toxicosis in swine: an overview of porcine pulmonary edema and current perspectives. Environ Health Perspect.

[39] Smith GW, Constable PD, Haschek WM (1996). Cardiovascular responses to short-term fumonisin exposure in swine. Fund Appl Toxicol.

[40] Marin DE, Gouze ME, Taranu I, Oswald IP (2007). Fumonisin B1 alters cell cycle progression and interleukin-2 synthesis in swine peripheral blood mononuclear cells. Mol Nutr Food Res.

[41] Marin DE, Taranu I, Pascale F, Lionide A, Burlacu R, Bailly JD (2006). Sex-related differences in the immune response of weanling piglets exposed to low doses of fumonisin extract. Br J Nutr.

[42] Johnson VJ, Sharma RP (2001). Gender-dependent immunosuppression following subacute exposure to fumonisin B1. Int Immunopharmacol.

[43] Bryden WL (2007). Mycotoxins in the food chain: human health implications. Asia Pac J Clin Nutr.

[44] Wild CP (2007). Aflatoxin exposure in developing countries: the critical interface of agriculture and health. Food Nutr Bull.

[45] Andretta I, Kipper M, Lehnen CR, Hauschild L, Vale MM, Lovatto PA (2012). Meta-analytical study of productive and nutritional interactions of mycotoxins in growing pigs. Animal.

[46] Prelusky DB (1996). A study on the effect of deoxynivalenol on serotonin receptor binding in pig brain membranes. J Environ Sci Health B.

[47] Bouhet S, Oswald IP (2005). The effects of mycotoxins, fungal food contaminants, on the intestinal epithelial cell-derived innate immune response. Vet Immunol Immunopathol.

[48] Oswald IP (2006). Role of intestinal epithelial cells in the innate immune defence of the pig intestine. Vet Res.

[49] Alassane-Kpembi I, Oswald IP, Nieworld T (2015). Effects of feed contaminants on the intestinal health of monogastric farm animals. Intestinal health: key to optimise production.

[50] Grenier B, Applegate TJ (2013). Modulation of intestinal functions following mycotoxin ingestion: meta-analysis of published experiments in animals. Toxins (Basel).

[51] Ghareeb K, Awad WA, Bohm J, Zebeli Q (2015). Impacts of the feed contaminant deoxynivalenol on the intestine of monogastric animals: poultry and swine. J Appl Toxicol.

[52] Bracarense AP, Lucioli J, Grenier B, Drociunas Pacheco G, Moll WD, Schatzmayr G (2012). Chronic ingestion of deoxynivalenol and fumonisin, alone or in interaction, induces morphological and immunological changes in the intestine of piglets. Br J Nutr.

[53] Bouhet S, Oswald IP (2007). The intestine as a possible target for fumonisin toxicity. Mol Nutr Food Res.

[54] Awad WA, Aschenbach JR, Setyabudi FM, Razzazi-Fazeli E, Bohm J, Zentek J (2007). In vitro effects of deoxynivalenol on small intestinal D-glucose uptake and absorption of deoxynivalenol across the isolated jejunal epithelium of laying hens. Poult Sci.

[55] Dietrich B, Neuenschwander S, Bucher B, Wenk C (2012). Fusarium mycotoxin-contaminated wheat containing deoxynivalenol alters the gene expression in the liver and the jejunum of broilers. Animal.

[56] Lessard M, Boudry G, Seve B, Oswald IP, Lalles JP (2009). Intestinal physiology and peptidase activity in male pigs are modulated by consumption of corn culture extracts containing fumonisins. J Nutr.

[57] Lalles JP, Lessard M, Boudry G (2009). Intestinal barrier function is modulated by short-term exposure to fumonisin B(1) in Ussing chambers. Vet Res Commun.

[58] Loiseau N, Debrauwer L, Sambou T, Bouhet S, Miller JD, Martin PG (2007). Fumonisin B1 exposure and its selective effect on porcine jejunal segment: sphingolipids, glycolipids and trans-epithelial passage disturbance. Biochem Pharmacol.

[59] Eriksen GS, Pettersson H (2004). Toxicological evaluation of trichothecenes in animal feed. Anim Feed Sci Technol.

[60] Pinton P, Oswald IP (2014). Effect of deoxynivalenol and other Type B trichothecenes on the intestine: a review. Toxins (Basel).

[61] Lucioli J, Pinton P, Callu P, Laffitte J, Grosjean F, Kolf-Clauw M (2013). The food contaminant deoxynivalenol activates the mitogen activated protein kinases in the intestine: Interest of ex vivo models as an alternative to in vivo experiments. Toxicon.

[62] Kolf-Clauw M, Castellote J, Joly B, Bourges-Abella N, Raymond-Letron I, Pinton P (2009). Development of a pig jejunal explant culture for studying the gastrointestinal toxicity of the mycotoxin deoxynivalenol: Histopathological analysis. Toxicol In Vitro.

[63] Yunus AW, Blajet-Kosicka A, Kosicki R, Khan MZ, Rehman H, Böhm J. Deoxynivalenol as a contaminant of broiler feed: intetsinal development, absorptive functionality, and metabolism of the mycotoxin. Poult Sci. 2012;91:852-61. 10.3382/ps.2011-01903 22399724

[64] Pierron A, Mimoun S, Murate LS, Loiseau N, Lippi Y, Bracarense AP (2016). Intestinal toxicity of the masked mycotoxin deoxynivalenol-3-beta-D-glucoside. Arch Toxicol.

[65] Pinton P, Nougayrede JP, Del Rio J-C, Moreno C, Marin DE, Ferrier L (2009). The food contaminant deoxynivalenol, decreases intestinal barrier permeability and reduces claudin expression. Toxicol Appl Pharmacol.

[66] Goossens J, Pasmans F, Verbrugghe E, Vandenbroucke V, De Baere S, Meyer E (2012). Porcine intestinal epithelial barrier disruption by the Fusarium mycotoxins deoxynivalenol and T-2 toxin promotes transepithelial passage of doxycycline and paromomycin. BMC Vet Res.

[67] Bouhet S, Hourcade E, Loiseau N, Fikry A, Martinez S, Roselli M (2004). The mycotoxin fumonisin B1 alters the proliferation and the barrier function of porcine intestinal epithelial cells. Toxicol Sci.

[68] Vandenbroucke V, Croubels S, Martel A, Verbrugghe E, Goossens J, Van Deun K (2011). The mycotoxin deoxynivalenol potentiates intestinal inflammation by Salmonella typhimurium in porcine ileal loops. PLoS One.

[69] Devriendt B, Gallois M, Verdonck F, Wache Y, Bimczok D, Oswald IP (2009). The food contaminant fumonisin B(1) reduces the maturation of porcine CD11R1(+) intestinal antigen presenting cells and antigen-specific immune responses, leading to a prolonged intestinal ETEC infection. Vet Res.

[70] Oswald IP, Desautels C, Laffitte J, Fournout S, Peres SY, Odin M (2003). Mycotoxin fumonisin B1 increases intestinal colonization by pathogenic *Escherichia coli* in pigs. Appl Environ Microbiol.

[71] Bouhet S, Le Dorze E, Peres S, Fairbrother JM, Oswald IP (2006). Mycotoxin fumonisin B1 selectively down-regulates the basal IL-8 expression in pig intestine: in vivo and in vitro studies. Food Chem Toxicol.

[72] Hoch RC, Schraufstatter IU, Cochrane CG (1996). *In vivo, in vitro,* and molecular aspects of interleukin-8 and the interleukin-8 receptors. J Lab Clin Med.

[73] Zachrisson K, Neopikhanov V, Wretlind B, Uribe A (2001). Mitogenic action of tumour necrosis factor-alpha and interleukin-8 on explants of human duodenal mucosa. Cytokine.

[74] Maheshwari A, Lacson A, Lu W, Fox SE, Barleycorn AA, Christensen RD (2004). Interleukin-8/CXCL8 forms an autocrine loop in fetal intestinal mucosa. Pediatr Res.

[75] Maresca M, Yahi N, Younes-Sakr L, Boyron M, Caporiccio B, Fantini J (2008). Both direct and indirect effects account for the pro-inflammatory activity of enteropathogenic mycotoxins on the human intestinal epithelium: stimulation of interleukin-8 secretion, potentiation of interleukin-1beta effect and increase in the transepithelial passage of commensal bacteria. Toxicol Appl Pharmacol.

[76] Cano PM, Seeboth J, Meurens F, Cognie J, Abrami R, Oswald IP (2013). Deoxynivalenol as a new factor in the persistence of intestinal inflammatory diseases: an emerging hypothesis through possible modulation of Th17-mediated response. PLoS One.

[77] Ali-Vehmas T, Rizzo A, Westermarck T, Atroshi F (1998). Measurement of antibacterial activities of T-2 toxin, deoxynivalenol, ochratoxin A, aflatoxin B1 and fumonisin B1 using microtitration tray-based turbidimetric techniques. Zentralbl Veterinarmed A.

[78] Burmeister HR, Hesseltine CW (1966). Survey of the sensitivity of microorganisms to aflatoxin. Appl Microbiol.

[79] Wache YJ, Valat C, Postollec G, Bougeard S, Burel C, Oswald IP (2009). Impact of deoxynivalenol on the intestinal microflora of pigs. Int J Mol Sci.

[80] Burel C, Tanguy M, Guerre P, Boilletot E, Cariolet R, Queguiner M (2013). Effect of low dose of fumonisins on pig health: immune status, intestinal microbiota and sensitivity to Salmonella. Toxins (Basel).

[81] Streit E, Schatzmayr G, Tassis P, Tzika E, Marin D, Taranu I (2012). Current situation of mycotoxin contamination and co-occurrence in animal feed--focus on Europe. Toxins (Basel).

[82] Alassane-Kpembi I, Puel O, Oswald IP (2015). Toxicological interactions between the mycotoxins deoxynivalenol, nivalenol and their acetylated derivatives in intestinal epithelial cells. Arch Toxicol.

[83] Grenier B, Oswald IP (2011). Mycotoxin co-contamination of foods and feeds: meta-analysis of publications describing toxicological interactions. World Mycotoxin J.

[84] Alassane-Kpembi I, Schatzmayr G, Taranu I, Marin D, Puel O, Oswald IP. Mycotoxins co-contamination: Methodological aspects and biological relevance of combined toxicity studies. Crit Rev Food Sci Nutr. 2016. In press. 10.1080/10408398.2016.1140632 26918653

